# Reversible severe glycogenic hepatopathy in type 1 diabetes

**DOI:** 10.1007/s10354-020-00795-6

**Published:** 2021-01-20

**Authors:** Jan de Laffolie, Clemens Kamrath, Diana Burchert, Claudia Böttcher, Stefan Alexander Wudy, Klaus-Peter Zimmer

**Affiliations:** 1 Dept of General Pediatrics and Neonatology, University Children’s Hospital, Justus-Liebig-Universität Gießen, Feulgenstr 12, 35392 Gießen, Germany; 2 Department of Pathology, Justus-Liebig-Universität Gießen, Gießen, Germany

**Keywords:** Hepatopathy, Diabetes, Glycogen, Metabolic control, Pediatric hepatology

## Abstract

**Case presentation:**

We report a case of severe glycogenic hepatopathy in a 17-year-old boy with poorly controlled type 1 diabetes. On presentation, major findings included unexplained pronounced hepatomegaly and increased liver enzymes, ferritin, and triglycerides. Histology and electron microscopy evaluation showed severe glycogen storage, steatosis, and signs of fibrosis, resembling the histomorphological findings of Mauriac syndrome. After information about the nature of the disease and intensification of insulin therapy with insulin pump, liver enzymes, ferritin, and triglycerides normalized within 1 month.

**Conclusion:**

Glycogenic hepatopathy is a rare but important potential complication in poorly controlled juvenile diabetic patients. With improved metabolic control, it is fully reversible.

## Introduction

Mauriac syndrome was first described in 1930 by Pierre Mauriac in children with poorly controlled type 1 diabetes with growth failure and delayed puberty, cushingoid features, hypercholesterolemia, and elevated liver transaminases [1]. While poor metabolic control in adolescents with type 1 diabetes is common, hepatomegaly and excessive glycogen storage is rare. The true incidence of Mauriac syndrome is not known, a possible genetic background in *PHKG2* (catalytic subunit of glycogen phosphorylase kinase) is described [2]. Improvement of metabolic control can reverse liver changes [3]. For the first time, electron microscope images of the liver are presented.

We report on a case of severe glycogenic hepatopathy in an adolescent male with type 1 diabetes. The characteristic histological changes reminded us of those seen in Mauriac syndrome in smaller children and resolved after intensive insulin pump therapy and improved treatment adherence.

## Case

**Fig. 1:**
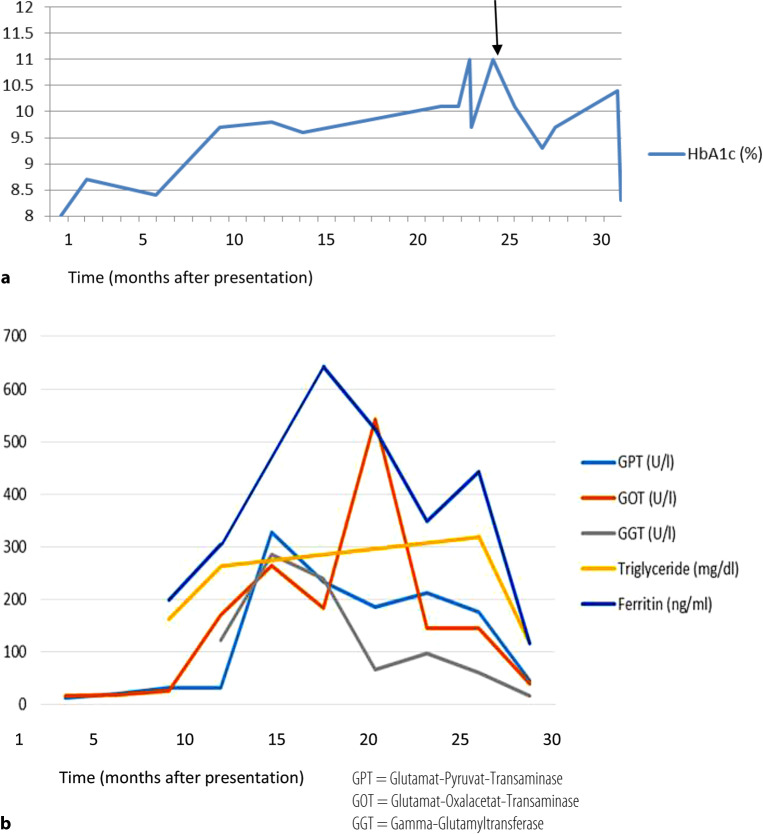
Visualization of major laboratory values by time in months from presentation (*arrow* signifies therapy intensification with family). **a** HbA1c over a period of 3 years during the episode; **b** transaminases over a period of 3 years during the episode. *GPT* Glutamat-Pyruvat-Transaminase, *GOT* Glutamat-Oxalacetat-Transaminase, *GGT* Gamma-Glutamyltransferase

A 17-year-old male patient with type 1 diabetes mellitus for 5 years developed increasingly worrisome HbA1c due to lack of adherence to the therapy (above 9%, max 11%). The attending physician observed an unexplained increase in serum transaminases, high ferritin and triglycerides, and hepatomegaly (Fig. 1). No other signs of diabetic microangiopathic complications were observed and there were no signs of pubertal delay, growth failure, cushingoid features, or obesity. There was also no sign of neutropenia, splenomegaly, high lactate. Frequent controls, psychotherapeutic interventions, and extensive information for the patient and his family did not improve HbA1c.

On ultrasound, the liver appeared coarse and hepatomegaly was persistently observed. Body mass index (BMI) was 20.7 kg/m^2^ (percentile 25–50). There was no family history of liver disease or metabolic disorders, no alcohol or tobacco use, and no drug consumption.

**Fig. 2:**
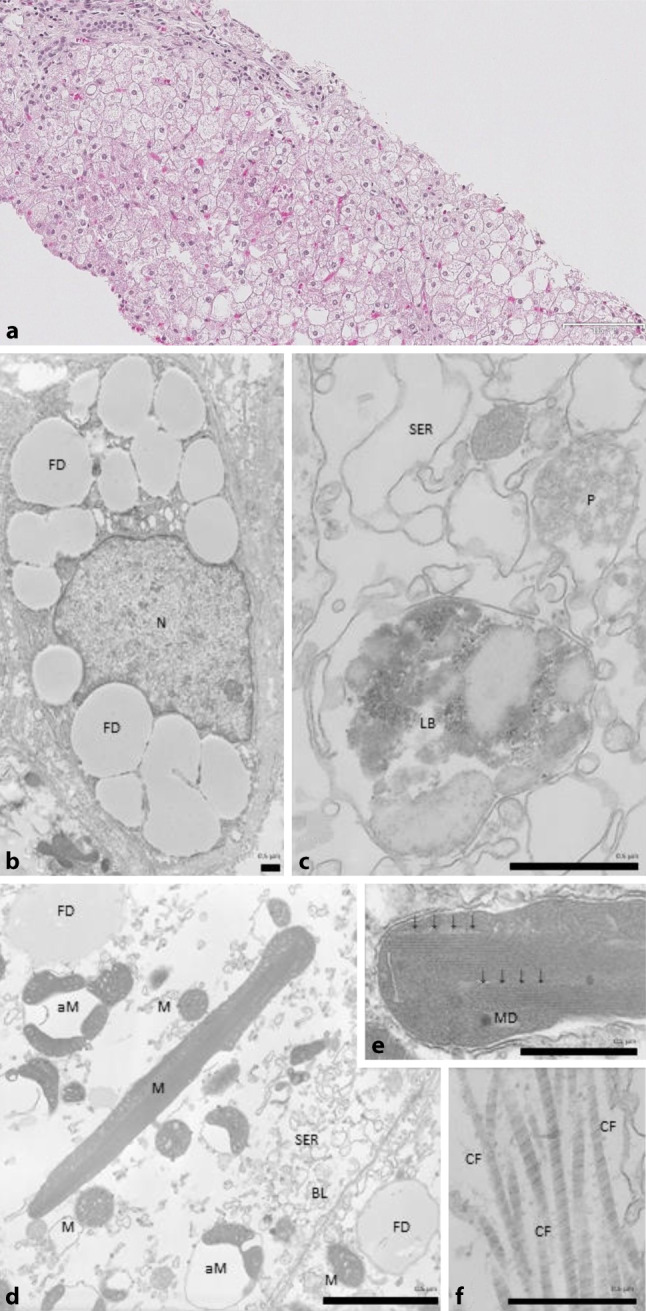
**a** Microscopic histology. Histology (Periodic acid-Schiff, magnification 20 ×) showed mixed macrovesicular steatosis in 10–20% of all hepatocytes, glycogenated nuclei consistent with morphological signs of glycogenosis type 1 or Mauriac syndrome. **b–f** Electron microscopy. Electron microscopic evaluation revealed Ito cells with glycogen phagocytosis, hepatocytes with polymorph, autophagic mitochondria, fat droplets, intracellular collagen fibers, and indirect signs of glycogenated nuclei. **b** Ito cell; **c** lipofuscin body of a hepatocyte; **d** pleomorphic mitochondria within a hepatocyte; **e** crystalloid inclusion (*arrows*) within a mitochondrion; **f** collagen formation within a hepatocyte. *N* nucleus, *FD* fat droplet, *LB* lipofuscin body, *P* peroxisome, *M* mitochondria, *aM* autophagic mitochondria, *MD* mitochondrial DNA, *CF* collagen fiber, *BL* basolateral membrane, *SER* smooth endoplasmic reticulum

Viral, autoimmune, and metabolic liver diseases were excluded. Biliary disease, coeliac disease, Wilson’s disease, hereditary hemochromatosis, drug-induced hepatotoxicity, and cystic fibrosis were ruled out. To exclude other causes of persistently elevated transaminases, a percutaneous liver biopsy was performed in analgosedation for histological and electron microscopic evaluation (Fig. 2).

For histology, routine biopsies were stained according to standards for pediatric liver histology including PAS (Periodic acid-Schiff) to improve glycogen visualization. Histology revealed mixed macrovesicular steatosis in 10–20% of all hepatocytes, glycogenated nuclei consistent with morphological signs of glycogenosis type 1 or Mauriac syndrome [4]. No signs of malignancy, fibrosis, or inflammation were present in histological staining.

For electron microscopy the tissue was embedded in epon and then cut using an ultramicrotome (Leica Microsystems, Germany). Thereafter, 55 nm cuts were transferred to Copper Nets (Plano, Germany) and leaded as described by Reynolds [5]. During this process, the glycogen is removed from the tissue, but indirect signs of glycogen storage can be observed.

On electron microscopic evaluation, hepatocytes were found with polymorph, autophagic mitochondria, fat droplets, intracellular collagen fibers, and indirect signs of glycogenated nuclei (Fig. 2). Interestingly, despite a lack of signs of fibrosis in Goldner staining, electron microscopy revealed signs of intracellular collagen synthesis.

In our patient insulin therapy was switched to an insulin pump and adherence was monitored closely. HbA1c decreased in parallel to the serum transaminases, ferritin, and triglycerides, back to normal values within 1 month (Fig. 1). After 6 months, liver size also declined slowly in sonography. Patient consent was obtained.

## Discussion

The clinical picture of Mauriac syndrome was not fulfilled completely, as the patient was well developed and did not show signs of growth failure, pubertal delay, or cushingoid facies [1]. Even in case of growth failure and pubertal delay, these could be attributed to poor glycemic control over a longer period.

Mauriac syndrome has become rare since pediatric diabetes management has continuously improved, but cases are still reported and the differential diagnosis needs to be considered in poorly controlled type 1 diabetes [6]. Most patients present with elevated HbA1c > 9% and symptoms resolve with improved control.

Some cases may be incorrectly labelled as non-alcoholic fatty liver disease (NAFLD), which can only be differentiated through biopsy and excessive hepatic glycogen storage.

The differential diagnosis of excessive glycogen storage includes glycogen storage diseases. Glycogen storage disease I (GSD I), also called von Gierke’s disease (described by Edgar von Gierke in 1929 [7]), is an inherited deficiency in glucose-6-phosphatase or glucose-6-phosphate translocase.

Without treatment, patients develop severe hepatomegaly, low blood glucose, and consecutive hyperlactatemia and hyperlipidemia. A subtype (GSDIb) will present with neutropenia and immunodeficiency. This was ruled out clinically in our patient, since glucose levels were rather high and type 1 diabetes was pre-confirmed.

To our knowledge, these are the first electron microscopic images of hepatocytes in pediatric hepatic glycogen storage disease resembling the morphological findings of Mauriac syndrome.

Fibrosis is not typically described in glycogen storage disease and is not part of descriptions of Mauriac syndrome in the literature, but the electron microscopic pictures (Fig. 2) give rise to speculations of early fibrotic changes and a much more severe course to be expected.

The etiology of Mauriac syndrome remains unclear; however, hypoinsulination and increased cortisolemia may play a role in increased glycogen storage [8–10]. Whether the pathomechanism is based on insulin deficiency or more on persisting hyperglycemia is still an important discussion and cannot be resolved in this patient.

In the meantime, genetic predispositions, e.g., by heterozygous mutations in genes of glycogen metabolism like glycogen phosphorylase kinase have been proposed [2]. In our patient, family history was negative for type 1 diabetes, glycogenosis, or fatty-liver disease. Genetic investigation was not performed as symptoms resolved with improved control.

In conclusion, it remains important to keep glycogenic hepatopathy in mind in diabetic patients with poor metabolic control. Thus, in these patients, NAFLD should not be assumed automatically. However, it is mandatory to rule out other forms of hepatopathy and to engage in intensified treatment to avoid further complications potentially leading to fibrosis, as found in electron microscopy in our patient.
